# Use of DEKOIS 2.0 to gain insights for virtual screening

**DOI:** 10.1186/1758-2946-6-S1-O24

**Published:** 2014-03-11

**Authors:** Frank M Boeckler, Matthias R Bauer, Tamer M Ibrahim, Simon M Vogel

**Affiliations:** 1 Department of Pharmacy & Biochemistry, Eberhard Karls University, Tuebingen, 72076, Germany

## 

With DEKOIS we have created an automated workflow to efficiently generate decoy sets based on a certain number of actives for any target [1]. Physico-chemical similarity should be maximized between decoys and actives in order to yield challenging sets for benchmarking, while exact mimicking of potentially active substructures should be avoided to omit latent actives in the decoy set (LADS). Overall, the diversity of actives and decoys should be maximized to avoid artifacts based on clusters. Applying this philosophy, we have added more details to describe the physicochemical space and applied this protocol to generate sets for targets which had not been accessible before. These DEKOIS 2.0 sets [2] are available online (http://www.dekois.com) for benchmarking and development of new tailor-made scoring functions. Further extension toward additional targets can facilitate a systematic comparison of the virtual screening performance of docking tools and scoring functions in a target dependent way. Based on DEKOIS 2.0, we have investigated strengths and weaknesses of popular docking tools and scoring functions. In addition, we assessed how differences in the setup and preparation can affect the pROC profiles and early enrichment, in particular [3]. For this analysis we have developed an automated protocol to identify and highlight chemotype-specific differences in ligand recognition.
